# Removal of Heavy Metal Ion Using Polymer-Functionalized Activated Carbon: Aspects of Environmental Economic and Chemistry Education

**DOI:** 10.1155/2020/8887488

**Published:** 2020-09-07

**Authors:** Hoang Thu Ha, Nguyen Thi Huong, Le Linh Dan, Nguyen Duy Tung, Vinh Bao Trung, Tran Dinh Minh

**Affiliations:** ^1^Faculty of Pedagogy, VNU University of Education, Vietnam National University, Cau Giay, Hanoi 100000, Vietnam; ^2^Faculty of Educational Management, VNU University of Education, Vietnam National University, Cau Giay, Hanoi 100000, Vietnam; ^3^Hanoi-Amsterdam High School for the Gifted, Hanoi, Vietnam; ^4^Department of Physics, High School for Gifted Students, Hanoi University of Science, Vietnam National University, Hanoi, Vietnam; ^5^High School of Education Sciences (HES), VNU University of Education, Vietnam National University, Cau Giay, Hanoi 100000, Vietnam; ^6^Faculty of Educational Technology, VNU University of Education, Vietnam National University, Cau Giay, Hanoi 100000, Vietnam

## Abstract

Numerous countries have shown signs of environmental pollution to prioritize economic growth and benefits, leading to seriously contaminated waters. This work indicated the method to synthesize a green material, which could remove contaminants to protect the natural environment. The porosity and functionality effects of amine-functionalized activated carbon (AFAC) enhanced the removal of toxic heavy metals (THMs) in aqueous solution. The raw activated carbon (RAC) was thermally modified with ultrahigh pure nitrogen (UHPN) at 500°C and 1000°C and then amine-functionalized with coupling agent of aminopropyltriethoxysilane (APS). They were denoted as AFAC-5 and AFAC-10, respectively. The data showed an enhanced metal adsorption capacity of the AFACs, because the modification produced more desired porosity and increased amine functional groups. AFAC-10, modified at a higher temperature, showed much higher THM adsorption capacity than AFAC-5, modified at a lower temperature, and RAC. The adsorption capacity decreased in the following order: Ni > Cd > Zn, which was in good agreement with the increasing electronegativity and ionic potential and the decreasing atomic radius. The maximum THM adsorption capacity of AFAC-10 for Ni, Cd, and Zn was 242.5, 226.9, and 204.3 mg/g, respectively.

## 1. Introduction

**Figure 1:**
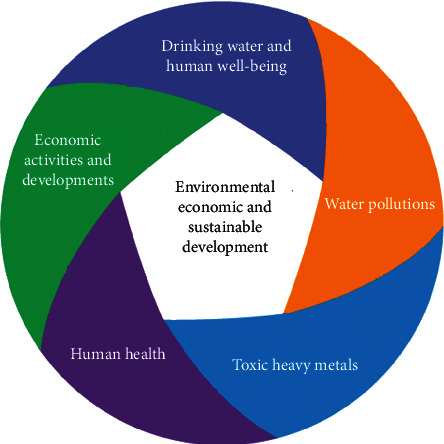
Socioeconomic implications of water pollution.

National economic development is a process of improving the physical and spiritual living conditions of that country through the production of property, improvement of social relations, and improvement of cultural quality [1–4]. However, due to the recent changes in the negative trend of the environment, it has raised the issue that timely solutions should be taken to move towards a sustainable economic development, in association with naturally environmental protection [5, 6]. Water is a valuable human resource but not permanent. Nowadays, the source of water for living and natural waters in developing countries, such as Vietnam, Laos, and Cambodia, is now increasingly polluted, especially heavy metal contaminations [7–10]. In particular, the water environment in many urban areas, industrial parks, and craft villages is increasingly polluted by wastewater, exhaust gas, and solid waste. For example, in these big cities, large industrial zones and hundreds of industrial production facilities are polluting the water environment due to the absence of facilities and waste treatment facilities.  Figure 1 shows the impact of socioeconomic development on the natural environment.

To develop the economy in tandem with protecting the environment and natural resources, the authors argue that they are not allowed to receive or limit the receipt of investors or investment projects that have caused harm to the ecosystem. The law maker in each country should only allow projects that have small effect on environmental pollution, projects that come with an advantaged technology for wastewater treatment. It is necessary to promote the development of green and environmentally friendly technologies/materials, such as those using renewable energy. Economic development should not be focused on exchanging environmental resources and environmental pollution. The location of projects also needs to be carefully considered in terms of geography, geopolitics, geopolitics, human well-being, and military, as appropriate for each country. At a rapid rate of industrialization, urbanization and population growth are the main causes of increasing pressure on water resources. Toxic heavy metals (THMs) accumulate in living organism and are not biodegradable [11]. Nickel and their compounds are distributed throughout various environments after their release from different sources such as Ni-Cd batteries, alloy manufacturing, printing, electroplating, and silver refining [12]. Nickel exposure is associated with increased risks of lung cancer, cardiovascular disease, neurological deficits, tightness of the chest, chest pain, and high blood pressure. Cadmium is mainly discharged into the environment from various industrial activities such as pigments, paints, textile dyes, metal smelting, paper manufacture, alloy, finishing, and thin film solar cells of Cd-Te industries [13]. The human health effects of Cd also include a number of acute and chronic disorders, such as “itai-itai” disease, emphysema, hypertension, liver, renal damage, and testicular atrophy. In general, zinc products are relatively nontoxic substances. However, excessive zinc uptake, even at low concentration, can give toxicity symptoms such as pale mucous membranes, nausea, vomiting, renal damage, cramps, pneumonitis, and fatigue.

Activated carbon (AC) is the most frequently used adsorbent material due to its displayed a desired porous structure and surface chemistry. To increase the adsorption capacity of metal ions, textural structure and surface properties of activated carbon were improved. In this study, we conducted the modification AC surface by heat treatment and polymer coating to in increase the desired functional group for THM adsorption. To our knowledge, until now, no work was examined to research the effect of the AC surface chemistry for THM adsorption. Therefore, it was demanded to investigate a reasonable procedure for the suitable AC types and enhanced a better understanding of the porosity and surface chemistry effects on THM adsorption from solutions.

## 2. Methodology

### 2.1. Preparation of AFACs

**Figure 2:**
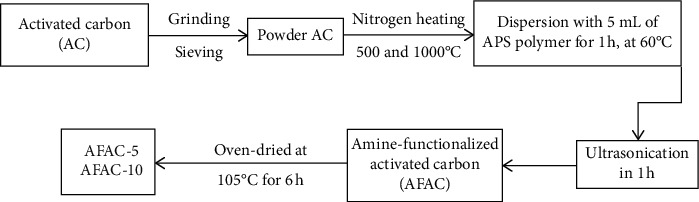
Synthesis pathway of adsorbents.

AC samples were purchased from Sigma Aldrich in the powder and decolorized. Before the experiments, the raw AC (RAC) was ground and sieved with the particle size around 500 *μ*m. 5 g of each RAC was heated at 500°C and 1000°C under ultrahigh purity nitrogen (UHPN) atmosphere in a furnace for 6 h. Afterward, the liquid-phase treatment was conducted by adding 5 mL aminosilane solution of aminopropyltriethoxysilane (APS, CAS Number 919-30-2, assay ≥ 98%), and the solution was gently ultrasonicated for 1 h at 60°C when the suspension was homogeneous. These products were washed with deionized water (DI water) several times and then oven-dried at 105°C for 6 h as precursors. The final material was denoted as AFAC-5 and AFAC-10 with different heating temperatures at 500°C and 1000°C, respectively (Figure 2).

### 2.2. Instruments

The innovation of green pathways to synthesize nanocomposite was of great importance for analytical chemistry as the impact of friendly pathways to natural environment is increasing within various analytical techniques for numerous material characterizations. Therefore, the pore morphology of all samples was determined through the Brunauer-Emmett-Teller (BET) method (ASAP/2020) to evaluate the specific surface area, pore volume, and diameter using N_2_ adsorption/desorption isotherm in the relative pressure (*P*/*P*_0_) range from 0.005 to 0.995. Prior to analysis, all the samples were degassed under vacuum overnight. The morphological features of AFACs were also observed using scanning electron microscopy (SEM) analysis (JEOL 5400) to identify the carbon physical surface. The amount of acidic and alkaline functional groups of samples was quantified by Boehm titration. Afterward, the materials were mixed with KBr and pressed into small discs prior to Fourier-transform infrared (FTIR/Bruker-Vector) analysis over the spectrum ranged of 4000–400 cm^−1^. The elemental analysis (EA) was characterized by using a PerkinElmer instrument. The inorganic compositions (minerals) and ash content were identified by using X-ray fluorescence spectroscopy (XRF-1800).

### 2.3. Batch Adsorption Experiments

The batch adsorption tests were performed by adding 30 mg AFACs and RAC into 30 mL solution of Ni^2+^, Cd^2+^, and Zn^2+^ in a concentration range of 30–400 mg/L. The suspension was stirred gently at 25°C and 120 rpm in a shaking water bath. After equilibration, the solid was settled down and filtered out by a membrane (Whatman No. 1 filter paper). The concentration of THM ions in the solution was measured by atomic absorption spectroscopy (AAS/AA-7000). The adsorption capacity *q*_*e*_ (mg/g) and efficient percentage (%R) of THM were, respectively, calculated as follows:  *q*_*e*_ =((*C*_0_  −  *C*_*e*_)  × *V* /*m*)  and % *R* = ((*C*_0_  −  *C*_*e*_)/*C*_0_)  × 100%, where *C*_0_ and *C*_*e*_ are the initial and equilibrium concentrations of THMs (mg/L); *m* is the amount of adsorbent (mg); and *V* is the volume of solution (L).

### 2.4. Adsorption Isotherms

The adsorption isotherm played an important role in the description how the metals interact with the adsorbent, and two models have used Langmuir and Freundlich equations as (*C*_*e*_/*q*_*e*_)= (*C*_*e*_/*q*_max_) + (1/*b* · *q*_max_) and ln *q*_*e*_ =  ln *K*_*F*_ + (1/*n*)ln *C*_*e*_, respectively, where, the Langmuir constant (*b*), described to the adsorption energy of the system, and the maximum adsorption capacity *q*_max_ (mg/g) are calculated from the slope and intercept of the linear plot of *C*_*e*_/*q*_*e*_ vs. *C*_*e*_. The Freundlich constant (*K*_*F*_) was associated with the bond energy, and the heterogeneity factor (1/*n*) displays the deviation from the linear part and is determined from the plot log (*q*_*e*_) vs. log (*C*_*e*_).

### 2.5. Adsorption Kinetics

The adsorption kinetic models were examined to describe the step which governs the THM adsorption onto materials. Two common equations were employed: pseudo-first-order and pseudo-second-order are given as ln(*q*_*e*_  −  *q*_*t*_) =  ln *q*_*e*_  −  (*k*_1_/2.303) *t* and (*t*/*q*_*t*_)=(1/*k*_2_*q*_*e*_^2^) + (1/*q*_*e*_)*t*, respectively, in which, *q*_*e*_ and *q*_*t*_ are, respectively, denoted as the adsorbed amount of metal ions at equilibrium (mg/g) and time *t* (min), and *k*_1_ is the rate constant (1/min). *q*_*e*_ and *k*_1_ are determined from the slope and intercept of the linear plot of ln (*q*_*e*_ − *q*) vs. *t*. And, *k*_2_ represents the equilibrium rate constant (g/mg·min).

## 3. Results and Discussions

### 3.1. Porosity Structure and EA Analysis

The EA and textural characterization results in Table 1 also confirmed that the thermal treatment and APS polymer dispersion strongly affected the BET surface area (BET), total pore volume (TVP), and mean pore diameter (MPD) of AFACs.

**Figure 3:**
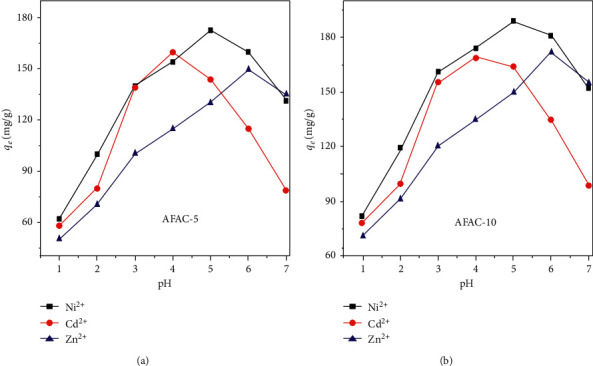
Effect of pH on the adsorption of THMs using (a) AFAC-5 and (b) AFAC-10.

RAC had a smaller BET specific surface area with a larger average pore width than the two AFACs. The pore volume and average pore width of AFAC-10 was reduced more than those in AFAC-5, which was attributed to the degradation of organic content (mainly O, N, and H) and the changes of surface heteroatoms, which could be reasonably associated with the weight loss experienced by the AFACs through the thermal and polymer coating procedure. AFAC-10 had the highest BET area (1407 m^2^/g), smallest TVP value (0.49 cm^3^/g), and smallest MPD (1.94 nm), which give it the largest quantity of wider micropores among the three samples. It is associated with the THM adsorption capacities (Figure 3); these superior morphological features of AFAC-10 afforded it with enhanced adsorption capacities at all metal initial concentration compared with AFAC-5 and RAC.

### 3.2. TEM Result

**Figure 4:**
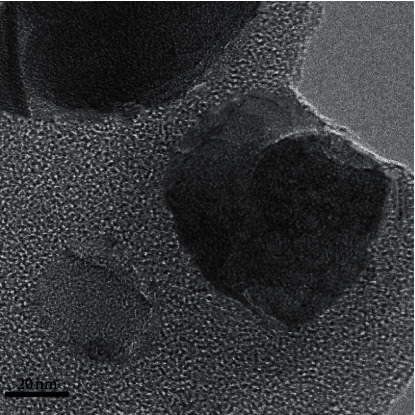
TEM image of AFAC-10.

These data in Table 1 also were consistent with the TEM image, where micropore structure was observed. As can be seen from Figure 4, it is rational to conclude that the larger micropores (1.96 nm) of AFAC-10 were not destroyed by polymer functionalization at high temperature.

### 3.3. Effect of Functionality

**Figure 5:**
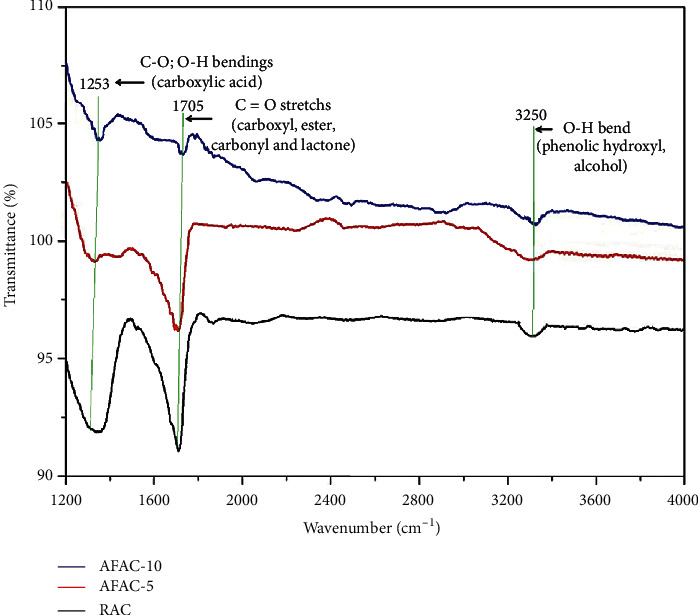
FTIR spectra result.

In order to identify the effect of these chemistry changes on the surface functionality of the three samples, FTIR analysis was conducted, and the results are interpreted in Figure 5.

Most of the intensive peaks in RAC are greater than those of the two AFACs. This indicates that the thermal treatment under UHPN condition significantly alleviated the total amount of O-bearing functional groups such as carboxyl, lactonel, phenol and carbonyl. In particular, the two strong peaks in RAC with high intensity at 1253 cm^−1^ were ascribed to single bond C-O and O-H bending of carboxyl groups. The peak at 1705 cm^−1^ was assigned to a double-bond C=O stretching of carboxyl, lactone, ester, and carbonyl groups. These three peaks were greater than those in the two AFACs. Furthermore, a fairly small peak at 3250 cm^−1^ assigned to O-H stretching in phenols and alcohols was degraded after the thermal modification. Consequently, the thermal modification alleviated the major O-containing functional groups.

### 3.4. Titration Studies

**Figure 6:**
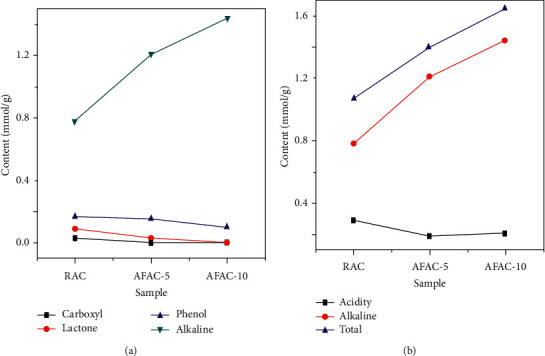
Functionality changes after the modifications.

Boehm's titration results shown in Figure 6 were used to determine the total concentration of acidity and basicity of all the AC samples. It indicated that the thermal treatment slightly reduced the total content of acidity, including O-bearing functional groups (phenol, carboxyl, and lactone). However, the alkalinity was greatly increased at the higher thermal treatment temperature (AFAC-10). The alkalinity contents of AFAC-5 and AFAC-10 were 1.2 and 1.32 mmol/g, which were 150 and 170% increased, respectively, compared with those of RAC. This thermal treatment-induced increase in alkalinity contributed to the enhanced THM removal capability of the AC THMs. This is similar to a previous report in which the amine groups showed an increasing trend for the removal of metal ions from wastewater.

### 3.5. Determination of pH_pzc_

**Figure 7:**
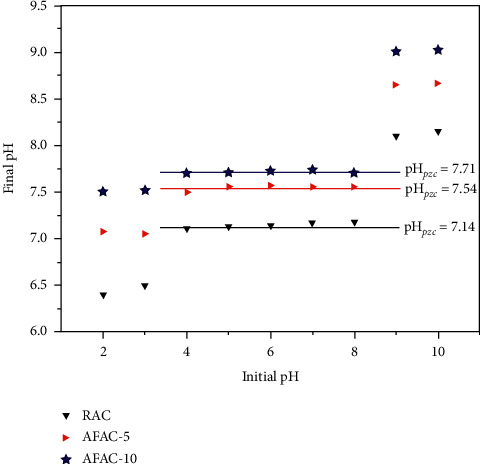
pH_pzc_ values of RAC, AFAC-5, and AFAC-10.

Zero point of charge, pH_pzc_, is an important parameter to determine the net surface charge density of materials in solution. The pH_pzc_ was calculated from the point of intersection of pH_initial_ vs. pH_final_ curve at the pH_initial_ = pH_final_, as shown in Figure 7. The pH_pzc_ values of RAC, AFAC-5, and AFTAC-10 were 7.14, 7.54, and 7.71, respectively. The pH_pzc_ of the two AFACs shows that they had more alkaline than the RAC due to the modifications. As pH_pzc_ is the pH at which the carbon net surface charge was zero, the surface of the AFACs has a negative charge at pH > pH_pzc_ but a positive charge at pH < pH_pzc_. Therefore, it is reasonable to say that the two AFACs had more net negative charge than RAC, which can contribute to reducing positive THM ions in aqueous solution.

### 3.6. Influence of pH

The optimum pH values for the removal of Cd^2+^, Ni^2+^, and Zn^2+^ ions using the two AFACs were 4.0, 5.0, and 6.0, respectively. The decrease in the ionic potential of THM Cd (2.11) > Ni (2.9) > Zn (2.7) increased the metallic solubility under specific pH conditions. In order to investigate the effects of modifications and solution pH on THM removal, 50 mg of AFAC-5 and AFAC-10 was added in 50 mL of THM ions at *C*_0_ of 200 mg/L, and the system mixture was agitated for 30 min. The maximum adsorption capacities of Cd^2+^, Ni^2+^, and Zn^2+^ by AFAC-5 were 159.81, 172.89, and 150.45 mg/g and by AFAC-10 were 168.85, 188.89, and 171.45 mg/g, at their optimal pH of 4.0, 5.0, and 6.0, respectively (Figure 3). The THM adsorption capacity of AFAC-10 was higher than that of AFAC-5 at all three THMs. At acidic conditions (pH < optimum pH), the increased concentrations of aqueous H^+^ ions competing with the THM ions on the adsorption sites of the modified AC surface substantially reduced the metal binding power on the AC surface. Thus, increasing pH is advantageous for metal sorption mainly because the elevated proportions of negative charges developed on the AC surface at acidic conditions (pH < optimum pH). Conversely, at higher pH (above optimum pH), THM cations might be precipitated in the solution due to the increased OH^−^ concentration, which can decrease the THM uptake capacity.

### 3.7. Dose Effect

**Figure 8:**
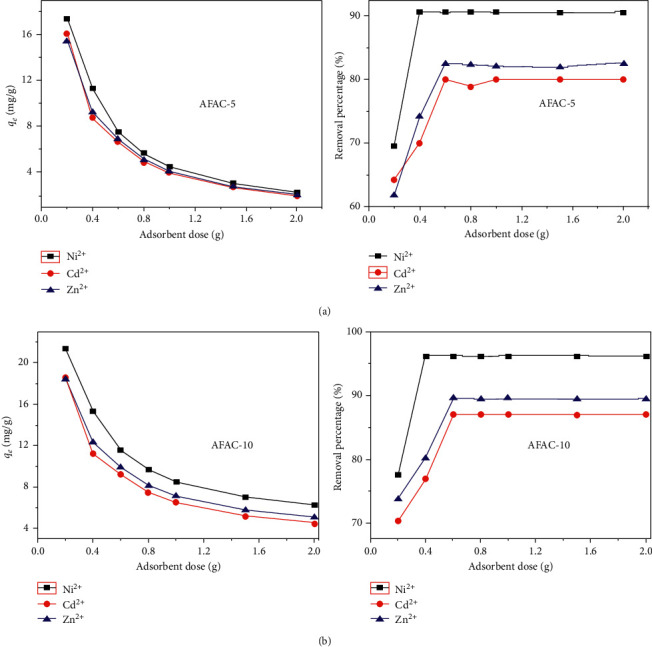
Dose effect on the sorption capacity (*q*_*e*_) and removal efficiency (%R) of AFAC-5 (a) and AFAC-10 (b).

As the weight of AFAC-5 and AFAC-10 increased from 0.2 to 0.8 g, the removal percentage increased for all three metals to maximums at 90.68, 82.5, and 80.01 and 96.23, 89.53 and 86.98% at optimum doses of 0.4, 0.6 and 0.6 g for Ni, Zn and Cd, respectively (Figure 8). The removal efficiency was increased with increasing adsorbent dose due to the increased surface area and active binding sites. The removal efficiency for the THMs under dose influence decreased in the following order: Ni > Zn > Cd.

Table 2 displays the decreases in the effective radii of hydrated ions and hydration energy enhanced the THM adsorption process, so that the highest adsorption capacity was observed with Ni^2+^, followed by Zn^2+^ and Cd^2+^. The removal efficiency of the metals was not significantly affected by further dose increase, which was ascribed to the aggregation or overlapping of adsorption sites caused by overcrowded adsorbent particles. This overlapping or aggregation may have reduced the total surface area available for THM adsorption per unit mass of the adsorbents and increased the diffusional path length, resulting in ineffective removal on adsorption capacity per unit mass of adsorbent.

### 3.8. Effect of Initial Concentration

**Figure 9:**
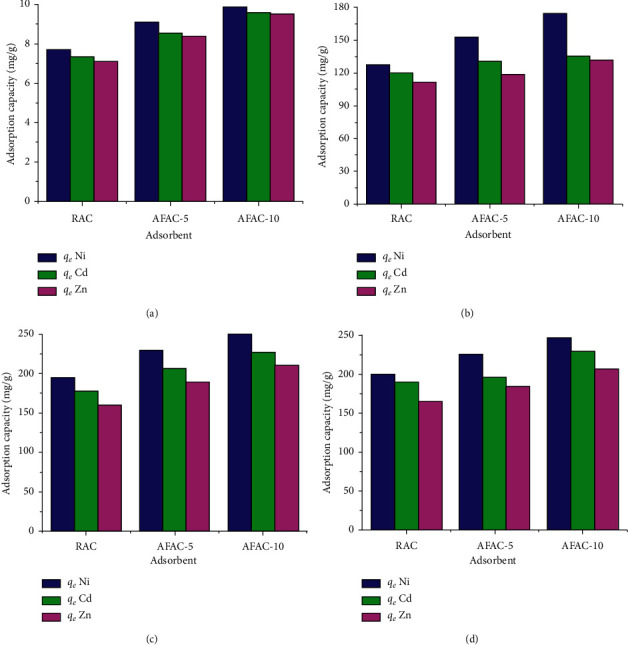
THM adsorption capacities by RAC, AFAC-5, and AFAC-10 at different *C*_0_ of (a) 10 mg/L, (b) 200 mg/L, (c) 400 mg/L, and (d) 600 mg/L.

Figure 9 shows the Ni^2+^, Zn^2+^, and Cd^2+^ adsorption capacities by the three samples at *C*_0_ of 10, 200, 400, and 600 mg/L under optimum conditions. As the initial concentration was increased from 10 to 400 mg/L, the removal capacities steadily increased but then only increased slightly as the THM concentration was further increased from 400 to 600 mg/L. This is because the amount of vacant or active sites available for occupation became saturated above 400 mg/L.

### 3.9. Removal Efficiency Comparison among the THMs

The metal adsorption capacity was highest for Ni^2+^, followed by Cd^2+^ and Zn^2+^, and decreased in the following order among the three ACs: AFAC-10 > AFAC-5 > RAC. These different affinities of THMs to the adsorbent surface were attributed to their chemical properties, such as metallic electronegativity, electron configuration, ionic potential, ion radius, and stability constants of metals, as shown in Table 2.

The decreasing THM adsorption capacity order (Ni > Cd > Zn) was in good agreement with the order of stability constants of metal-humic and -fulvic acid complexes, as previously reported. The adsorption capacity order was also the same as the decreased degree of Pauling electronegativity for Ni, Cd, and Zn of 1.91, 1.69, and 1.65, respectively. Allen and Brown found that the more metallic electronegativity could be more strongly attracted to the surface [17]. The adsorption capacity of the THMs also correlated with the metal ion atomic size: increasing with decreasing atomic ion radius (Table 3). Metals with smaller ionic size have enhanced sorption onto carbon-fixed specific surface area. Furthermore, the metal sorption diminished with increasing ionic potential of Ni (2.9) and Cd (2.11). The difference in IR absorption bands of the ACs before and after the metal sorption also supports the different adsorption power among the three THMs. After the metal adsorption, the IR absorption bands were shifted to longer wavelength, which indicates the binding of the sorption sites with the THMs, as also confirmed by the adsorption band shift in the IR spectra (*δ*) before and after the THM adsorption processes (Table 4). The largest band shift was observed in Ni^2+^ adsorption, followed by Cd^2+^ and Zn^2+^.

These shift changes indicate that the metal binding processes occurred on the adsorbent surface. In general, a greater shift change (Δ*δ*) indicates a stronger energy between adsorbent-adsorbate interactions.

### 3.10. Equilibrium Studies

The Langmuir and Freundlich isotherms were investigated for their ability to describe the adsorption behavior of divalent metals on the adsorbents. The experimental result listed in Table 5 shows the THM adsorption data were better fitted with the Langmuir isotherm model than with the Freundlich isotherm, having a high determination coefficient (*R*^2^) of 0.99, 0.988, and 0.987 for Cd^2+^, Ni^2+^, and Zn^2+^, respectively.

The obtained parameter results suggested that the metal adsorption behavior on AFAC-10 is monolayer adsorption with homogeneous distribution of active sites on the surface. All values of 1/*n* obtained from the Freundlich isotherm also suggest that the adsorption process on the sorbent is favorable for these divalent metals.

Table 6 compares the maximum adsorption capacities of the three adsorbents for the three metals. The maximum adsorption capacities of Ni, Cd, and Zn of AFAC-10 calculated from the Langmuir isotherm model were 242.5, 226.9, and 204.3 (mg/g), respectively, which were higher than previously reported values (Table 6). This result indicates that AFAC-10 might be used as a promising adsorbent for metal removal from wastewater.

### 3.11. Kinetics

**Figure 10:**
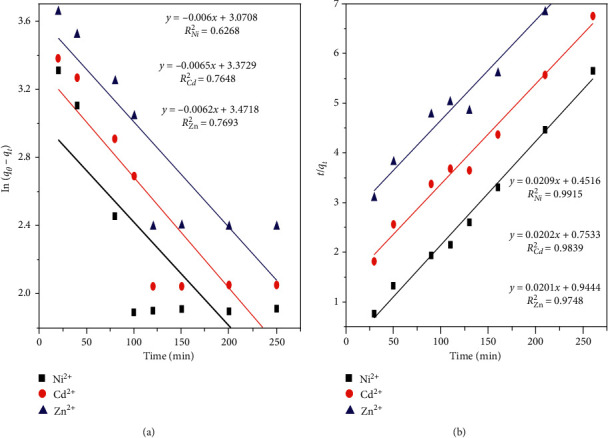
Kinetic curves fitting the adsorption process by AFAC-10. (a) Pseudo-first-order-plot. (b) Pseudo-second-order-plot.

In order to verify the kinetics of metal adsorption behavior, pseudo-first-order and pseudo-second-order kinetic models were applied to the experimental data. The adsorption parameters and the determination coefficients are shown in Figure 10 and Table 7, respectively.

The experimental results for the maximum capacities (*q*_*e*,exp_) of Ni^2+^, Cd^2+^, and Zn^2+^ (43.06, 46.02, and 47.61 mg/g, respectively) better matched the calculated values (*q*_*e*,cal_) (47.84, 49.5, and 49.85 mg/g, respectively) in the pseudo-second-order model, which showed a higher determination coefficient (*R*^2^) (around 0.99, 0.98, and 0.97, respectively). This indicates that the metal adsorption can be represented by chemisorption occurring during the surface interaction. Thus metals are removed or favorably adsorbed by chemical interactions with functional groups existing on the surface of the adsorbents. The rate of adsorption is proportional to the area of the amount of available sites. This adsorption process has a second-order, not first-order, model mechanism, based on the assumption that the rate-limiting step is chemisorption, involving valence forces through the ion exchange of electrons between adsorbate-adsorbent bonds.

### 3.12. Chemistry Education Research and Practice via Active Learning in Schools

**Figure 11:**
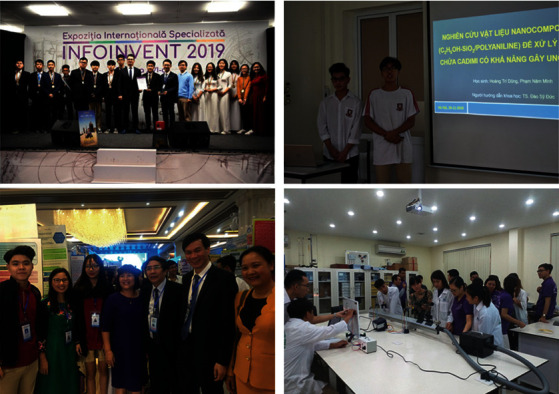
Chemistry education via STEAM teaching in HES and UEd.

Vietnam's education is facing development requirements to meet the task of industrialization, modernization of the country, and successful international integration. Therefore, the mission of the education sector is to constantly improve the quality of training and the effectiveness of scientific research, in order to contribute to training high-quality labor resources for the country. Along with the development of knowledge, workers must have the essential skills to adapt to a dynamic and diverse social context and environment. Innovating teaching methods is one of the urgent requirements for the education sector to improve the quality of education and training, the survival of each training institution. Active learning in schools increases the student ability in science, technology, engineering, art, and mathematics (STEAM). According to the current trend of higher education development in the direction of “capacity training for students,” the ability to practice experiments is one of the most important competencies students need to develop when studying natural science subjects. The reality of applying experiments in teaching and learning natural science subjects at universities is still not really effective, in addition, materials (including textbooks, handouts, and course books) for teaching and learning practice experiments in the direction of STEAM approach in natural science subjects. Especially for chemistry, they are very limited, and there is lack of experts and specific lesson plans for students to practice laboratory experiments. Therefore, this work needs to contribute to helping teachers to teach natural science subjects at university more effectively, making students more interested in experimenting and researching science. The research group of cofirst author Dr. Tran Dinh Minh in University of Education (UEd), Vietnam National University, have conducted to evaluate the ability of integrated scientific activities via STEAM-practical application in improving UEd students to have better knowledge. The chemistry experimental design textbook and course book are part of the school's advanced chemistry and science curriculum. The group also builds up the STEAM teachers toward practicing and designing the STEAM activities in UEd. The HES and UEd teachers and students have participated in many national and international competitions and had won several medals (Figure 11) [27–29].

Nowadays, integrated education via STEAM gained much attraction from scientists and publications. Our teachers need to understand STEAM-practical education, which critically shifts students with the necessary knowledge and skills associated with the fields of science, technology, engineering, and chemistry. These knowledge and skills have to be integrated and complementary to help students not only to well-understand the principles but also to apply to practice in real life as well as create products in their daily lives. STEAM-practical education can also be implemented through scientific research at UEd with a variety of topics in areas such as environmental chemistry, nanochemistry, renewable energy, and environmental protection, climate change, and high-tech agriculture. Experimental activities for students who are competent, interested, and passionate about exploration activities, scientific discoveries, and techniques for solving practical problems in daily life are a very important issue. STEAM-teachers need to organize well-organized science and technology activities in order to implement research projects within the framework of the annual science and technology competition for students at UEd. In the current context of technology, undergraduate and graduate programs of the VNU-UEd need to apply new technologies in teaching and experimenting to train in a class. The STEAM teacher has good professional knowledge and at the same time has many good technology skills, including skills and thinking. Scientific research is an important activity of higher education, one of the key measures to improve the quality of training, a combination of human resource training, talent fostering, and a good form of self-training for both teachers and students at the university.

**Figure 12:**
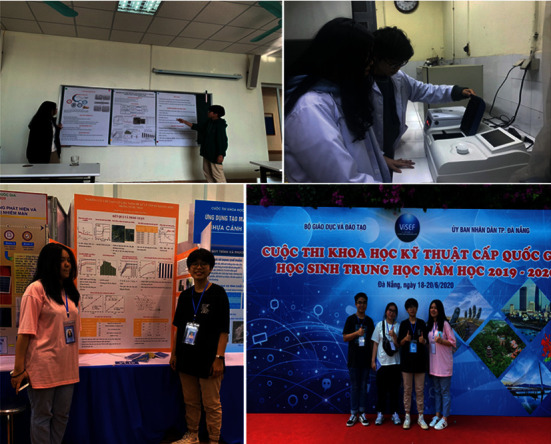
Practical chemistry education in schools.

There are many teaching methods approaching STEAM, but for chemistry, a very effective and positive method is to use the method of using visual experiments. Researching the latest technologies, experiments for students to apply software in teaching based on specific laboratory research models as well as international links contribute not only opening up new research directions but also valuable reference materials for lecturers, especially graduate teachers teaching new training programs of UEd (Figure 12).

## 4. Conclusions and Discussion

The present metal adsorption study of AFACs afforded the following conclusions:

- The thermal treatment and polymer dispersion of ACs decreased the oxygen-containing functional groups, the pore volume, and the size but increased the alkaline (amine) groups and BET surface area.
- AFAC-5 and AFAC-10 had more wide micropores (mean diameter 1.96 nm in AFAC-10) and more narrow mesopores (mean diameter 2.43 nm in AFAC-5) and exhibited an enhanced THM adsorption capacity and are thus promising materials for application to metal cation adsorption processes.
- The THM adsorption capacity of the AFAC-5 and AFAC-10 decreased in the following order: Ni > Cd > Zn, which corresponds with the order of metal chemical properties, such as increasing electronegativity, stability constants of metal-humic and -fulvic acid complexes, and decreasing ion potential and atomic radii.

## Figures and Tables

**Table 1:** Surface chemistry variations and weight of carbon samples (wt%).

Sample	BET (m^2^/g)	TVP (cm^3^/g)	MPD (nm)	Weight (g)	C (wt%)	H (wt%)	O (wt%)	N (wt%)
RAC	1029	0.96	3.38	2.11	74.93	0.78	3.02	0.85
AFAC-5	1227	0.75	2.50	1.95	86.38	0.60	1.90	4.34
AFAC-10	1407	0.49	1.94	1.61	94.56	0.48	0.73	4.25

**Table 2:** Metal affinity order of Ni, Cd and Zn.

Properties	Ni	Zn	Cd
^*a*^Hydrated cation radii (Å)	4.04	4.30	4.26
^*b*^Hydration energy (kJ/mol) (ΔG_h_)	−1980	−1955	−1755

^*a*^[14, 15]; ^*b*^[16].

**Table 3:** Ionic properties of Ni, Cd, and Zn.

Characteristics	Ni	Cd	Zn
Stability constant of metal-humic acid complexes^*a*^	3.2	2.78	2.74
Stability constant of metal-fulvic acid complexes^*b*^	3.1/3.2	—	2.4/2.3
Electronic configuration	3d^8^4s^0^	4d^10^5s^0^	3d^10^4s^0^
Pauling electronegativity^*c*^	1.91	1.69	1.65
Atomic radii^*c*^ (pm)	69	95	—
Ionic potential^*c*^	2.9	2.11	—

^*a*^[18]; ^*b*^[19]; ^*c*^[20].

**Table 4:** IR spectra of metals before and after adsorption.

Metal	Intensities of infrared absorption bands		
	Virgin metal	Adsorbed metal	Δ*δ*
Ni	735.49	804.81	69.32
Cd	762.08	827.93	65.85
Zn	1020.39	1075.52	55.13

**Table 5:** Isotherm model parameters.

Metal	Langmuir model			Freundlich model		
	*q*_max_ (mg/g)	*b*	*R* ^2^	*K*_*F*_	1/*n*	*R* ^2^
Ni^2+^	242.5	0.0237	0.9885	10.711	0.5555	0.9565
Cd^2+^	226.9	0.0215	0.9911	7.942	0.5791	0.9347
Zn^2+^	204.3	0.0141	0.9884	5.631	0.6105	0.9529

**Table 6:** Comparison of sorbents for metal removal from wastewater.

Adsorbate	Adsorbent	*q*_*m*_ (mg/g)	References
Ni	Orange peel	158.0	[21]
	Kaolinite	167.0	[22]
	AFAC-10	242.5	This study

Cd	Silica composite	166.7	[23]
	Sugarcane bagasse	189.0	[24]
	AFAC-10	226.9	This study

Zn	Macroalga	128.8	[25]
	Sludge	168.0	[26]
	AFAC-10	204.3	This study

**Table 7:** Pseudo-first-order and pseudo-second-order values.

Metal	*q*_*e*,exp_ (mg/g)	Pseudo-first-order model			Pseudo-second-order model			
		*k*_ad_ (min^−1^)	*q*_*e*,cal_ (mg/g)	*R* ^2^	*k* (g mg^−1^·min^−1^)	*q*_*e*,cal_ (mg/g)	*R* ^2^	*h* (mg g^−1^·min^−1^)
Ni	43.06	0.00598	21.55	0.6268	0.00097	47.84	0.9915	2.2143
Cd	46.02	0.06448	29.16	0.7648	0.00054	49.50	0.9839	1.3275
Zn	47.61	0.00621	32.19	0.7693	0.00043	49.75	0.9748	1.0589

*q*_*e*,cal_: calculated maximum adsorption capacity; *q*_*e*,exp_: experimentally determined maximum adsorption capacity.

## Data Availability

All the data and supporting materials are included within the article.
